# Axial involvement in enthesitis-related arthritis: results from a single-center cohort

**DOI:** 10.1186/s12969-023-00792-0

**Published:** 2023-02-06

**Authors:** Yanli Guo, Yuying Fang, Tonghao Zhang, Yuting Pan, Panpan Wang, Zhidan Fan, Haiguo Yu

**Affiliations:** Department of Rheumatology and Immunology, Children’s Hospital of Nanjing Medical University, Nanjing, 210008 China

**Keywords:** Enthesitis-related arthritis, Axial, Biologics, PRINTO, Disease-modifying anti-rheumatic drugs

## Abstract

**Background:**

Axial involvement in children with enthesitis-related arthritis (ERA) has characteristics that differ from those of peripheral involvement. This study characterized their clinical characteristics and treatment.

**Methods:**

Patients with ERA at the Children’s Hospital of Nanjing Medical University between January 2018 and December 2020 were included. The ERA cohort was divided into two based on the presence or absence of axial joint involvement. Demographic characteristics, clinical features, and treatments were described and compared.

**Results:**

In total, 105 children with ERA were enrolled (axial ERA, *n* = 57; peripheral ERA, *n* = 48). The age at disease onset of the axial group tended to be higher (11.93 ± 1.72 *vs.* 11.09 ± 1.91 years) and the diagnosis delay was bigger in patients with axial ERA (10.26 ± 11.66 months *vs.* 5.13 ± 7.92 months). The inflammatory marker levels were significantly higher in patients with axial. There were no differences in HLA-B27 positivity between the groups (34 [59.65%] *vs.* 28 [58.33%], *P* > 0.05). Hip involvement was more frequent in the axial group (52.63% vs 27.08%; X^2^ = 7.033). A total of 38 (66.67%) and 10 (20.83%) patients with axial and peripheral ERA, respectively, were treated with biological disease-modifying anti-rheumatic drugs (DMARDs) at diagnosis. The administration of biologics increased gradually in the axial ERA group, peaking at 18 months and decreasing thereafter, whereas that in the peripheral ERA group peaked at 6 months and began to decline thereafter.

**Conclusions:**

Axial ERA is a persistent active disease and requires a more aggressive treatment. Classification and early recognition of axial involvement may help with timely diagnosis and appropriate management.

## Background

Enthesitis-related arthritis (ERA) is a category of juvenile idiopathic arthritis (JIA) [1]. Studies have reported that ERA accounts for at least one-third of their JIA cases among Chinese populations [2, 3]. As an inflammatory arthritis highly associated with HLA-B27 in children, enthesitis and involvement of peripheral and axial joints are the typical features of this disorder [4, 5]. Patients with ERA tend to have greater pain intensity, higher disease activity, and poorer arthritis outcomes than those with other types of JIA [6]; additionally, it is more difficult for patients with ERA to achieve and maintain clinical and radiological remission than for those with other types of JIA [7].

ERA is considered a distinct disease entity from adult ankylosing spondylitis (AS), with peripheral joint involvement and entheseal disease common at presentation and axial involvement as a late feature. However, increased utilization of magnetic resonance imaging in detecting silent and early sacroiliac disease has shown that axial involvement is more common and presents earlier than previously thought in ERA [8]. With no objective signs of axial inflammation, occasional asymptomatic presentation, and ineffectiveness of disease-modifying anti-rheumatic drugs (DMARDs) for the condition, it is not surprising that the overall prognosis for axial involvement in ERA is poor. Compared with the peripheral phenotype, the axial phenotype of ERA may require a different approach to preventing progressive damage. Since ERA has a relatively poor outcome and axial disease even more so, the distinction between phenotypes is important to highlight. The International League Against Rheumatism (ILAR) did not recognize the two ERA subgroups, but the new Pediatric Rheumatology International Trials Organization (PRINTO) proposal does [1, 9]. Unfortunately, there is little evidence that might be used to compare the clinical characteristics between children with axial and peripheral ERA. Thus, this study aimed to describe and compare the clinical, laboratory, and treatment characteristics of the two distinct subtypes of ERA accumulated over 2 years pre-pandemic from one site in China.

## Methods

This study included patients diagnosed with JIA and ERA at the Children’s Hospital of Nanjing Medical University, China, between January 2018 and December 2020. Patients with ERA were followed up at least four times for 24 months. The diagnosis of JIA and ERA was based on the ILAR criteria [1]. Information about demographic and clinical features, laboratory data, and imaging and treatment at disease onset and thereafter at the 6-, 12-, 18-, and 24-month follow-up were recorded and analyzed.

This study was approved by the Ethics Committee of Children's Hospital of Nanjing Medical University (202008041–1).

Referred to the ASAS criteria, patients with ERA were classified into axial and peripheral groups based on arthritis involvement [10]. We defined axial disease by the presence of at least one criteria among: 1) inflammatory low-back pain or inflammatory dorsal pain lasting for more than 1 month; 2) limited spine mobility, as defined by a Schober index < 10 + 4 cm; 3) sacroiliac pain at examination sacroiliac pain at examination or alternating buttock pain; or 4) presence of axial disease by any available radiological examination [11]. Sacroiliitis was defined as sacroiliac pain at examination, alternating buttock pain, or evidence of inflammation (e.g., bone marrow edema, joint space enhancement, or erosions/sclerosis) on MRI [12, 13]. The presence of axial arthritis can be assessed by clinical and laboratory assessments and presence of inflammation on MRI of sacroiliac joints and spine. Those with only appendicular skeletal involvement were classified as having peripheral disease. Active joints were defined as joint swelling or, in the absence of swelling, a limited range of motion along with tenderness. Enthesitis was defined as chronic inflammatory pain or tenderness at the insertion site of the principal tendon, ligament, capsule, or fascia into the bone. Sacroiliitis was defined as sacroiliac pain at examination, alternating buttock pain, or evidence of inflammation (e.g., bone marrow edema, joint space enhancement, or erosions/sclerosis) on MRI [12, 13].

The demographic details, personal and family history of inflammatory and auto-immune disease or psoriasis, age at disease onset, time between onset of symptoms and diagnosis, number and site of onset joints, medication history, and laboratory data on disease onset such as C-reactive protein (CRP), erythrocyte sedimentation rate (ESR), HLA-B27, antinuclear antibody (ANA), and rheumatoid factor (RF) were recorded for all patients. All patients underwent regular screening for uveitis by an ophthalmologist. The medications used by each patient, including nonsteroidal anti-inflammatory drugs (NSAIDs), conventional DMARDs (such as methotrexate [MTX] and sulfasalazine), and biologic therapy with tumor necrosis factor (TNF)-α blockade (such as adalimumab, etanercept, and infliximab), were reviewed and recorded.

## Statistical analysis

Statistical analysis was performed using SPSS Statistics software (version 23; IBM Corp, Armonk, NY). Categorical data were expressed by counts or percentages, and compared between different groups using the chi-squared test or continuity correction as appropriate. The continuous variables were presented as mean (standard deviation). The variables were investigated using visual methods (descriptive statistics, probability plots) to determine whether they were normally distributed. Comparisons between the groups were performed using the chi-square test or Fisher’s exact test for qualitative variables and Student’s t-test for quantitative variables. P-values < 0.05 were considered significant.

## Results

### Distribution of JIA

**Fig. 1:**
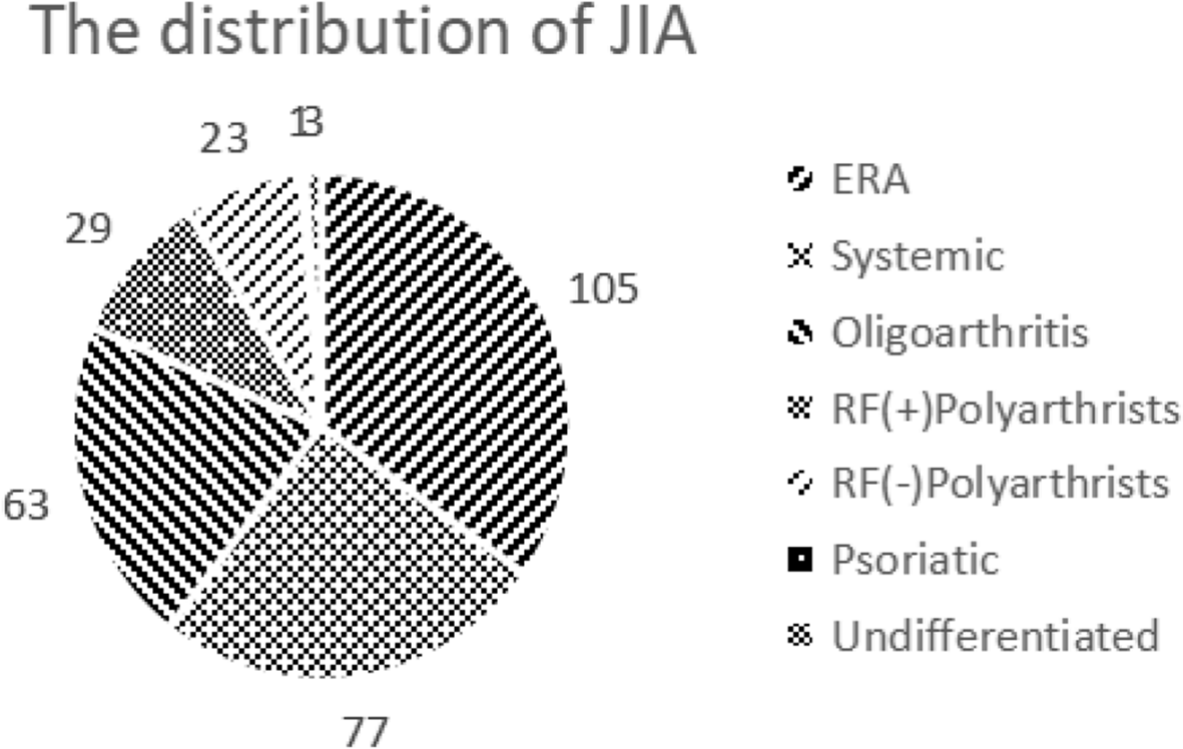
The distribution of JIA

A total of 301 children with JIA were included in the study (Fig. 1); these subjects had been previously diagnosed according to the classification criteria of the ILAR [1]. ERA (105, 34.88%) was the largest single type of JIA, while psoriatic (1, 0.33%) and undifferentiated JIA (3, 0.1%) were the least common types. A total of 105 children fulfilled the criteria for ERA at the time of diagnosis and were enrolled in this study; of these, 57 (54.29%) had axial ERA and 48 (45.71%) had peripheral ERA.

### Patient characteristics

The baseline characteristics of the patients diagnosed with ERA are shown in Table 1. In the ERA group, there was male predominance (88.57%) at a ratio of 7.75:1. The mean ± SD age at diagnosis for the combined group was 11.55 ± 1.85 years (axial ERA group, 11.93 ± 1.72 years; peripheral ERA group, 11.09 ± 1.91 years) (*P* < 0.05). The diagnosis delay was longer in patients with axial ERA (10.26 ± 11.66 months) than in those with peripheral ERA (5.13 ± 7.92 months) (*P* < 0.05). HLA-B27 was reported in 62 (59.05%) of the total 105 children (axial ERA group, 59.65%; peripheral ERA group, 58.33%; *P* > 0.05). A total of 29.52% of the patients had a family history of HLA-B27-related diseases in a first-degree relative. Uveitis was only reported in 5 (4.76%) of the patients, and no significant differences with respect to uveitis or family history were observed between the groups. Eight patients had fever, including six with axial ERA and two with peripheral ERA. Sex distribution and proportion of patients with fever were similar in both groups.

**Table 1:** Baseline characteristics of patients diagnosed as enthesitis-related arthritis at onset

Characteristic	Axial ERA (*n* = 57)	Peripheral ERA (*n* = 48)	*P*
Age at diagnosis, mean ± SD years	11.93 ± 1.72	11.09 ± 1.91	0.019*
Time between onset of symptoms and diagnosis, mean ± SD months	10.26 ± 11.66	5.13 ± 7.92	0.006*
Male sex, n (%)	49 (85.96)	44 (91.67)	0.360
HLA-B27 presence, n (%)	34 (59.65)	28 (58.33)	0.891
A family history positive for HLA-B27 related diseases, n (%)	16 (28.07)	15 (31.25)	0.722
Uveitis, n (%)	2 (3.51)	3 (6.25)	0.844

*P＜0.05 is statistically significant

### Laboratory variables at ERA onset

With respect to laboratory values such as white blood cell count, ANA, and RF, no significant differences were observed between the groups (Table 2). Regarding the disease activity measures, although no significant differences were found in the total serum CRP levels, CRP were upregulated in 15 (26.32%) patients with axial and 5 (10.42%) in peripheral ERA. There were significant differences in up-regulated CRP levels and ESR, and the inflammatory marker levels were significantly higher in patients with axial ERA than in those with peripheral ERA.

**Table 2:** Laboratory variables of enthesitis-related arthritis at onset

Laboratory variables	axial ERA (*n* = 57)	peripheral ERA (*n* = 48)	*P*
Hb (110–160)	127.07 ± 12.14	129.1 ± 9.79	0. 353
PLT (100–300)	314.15 ± 78.26	294.60 ± 58.15	0.131
WBC (4–10)	8.04 ± 1.85	8.08 ± 2.47	0.917
CRP (0–10)	16.307 ± 15.21	13.03 ± 10.61	0.199
ESR (≤ 20)	33.40 ± 15.04	24.52 ± 9.72	0.000*
ALT (7–30)	12.12 ± 7.07	14.38 ± 10.20	0.186
AST (14–44)	19.25 ± 7.97	20.23 ± 6.31	0.491
Alb (39–54)	45.70 ± 3.79	46.11 ± 3.56	0.572
CD4	822.76 ± 327.01	636.45 ± 266.84	0.030*
CD8	638.07 ± 234.49	571.64 ± 315.02	0.370
ANA, n (%)	9 (15.79)	9 (18.75)	0.688

*P＜0.05 is statistically significant

### Clinical distribution of articular involvement at ERA onset

Enthesitis was present in 65 (61.90%) patients (Table 3). The hip, knee, and ankle were the most common joints involved in active lower limb arthritis. Patients with axial ERA had more hip arthritis as well as sacroiliac and lumbar spine symptoms. Peripheral arthritis was present in 53 (92.98%) patients. No difference in distribution was observed for the shoulder, elbow, wrist, knee, ankle, or midfoot when patients with and without axial involvement were compared. Among the patients with axial disease, hip arthritis and cervical spine and lumbar vertebral involvement were observed in 30, 6, and 7 patients, respectively. Hip joint involvement occurred in over half of patients with axial ERA (52.63%), which was significantly more frequent than that in patients with peripheral ERA. Sacroiliitis was observed in all 57 patients with axial ERA and 18 (31.58%) patients had asymptomatic inflammation in the sacroiliac joint, as demonstrated on MRI. Enhancement, subchondral bone marrow edema, cartilage abnormalities, periarticular erosions, subchondral fatty marrow infiltration, and ankylosis were the main MRI findings of symptomatic sacroiliitis, while bone marrow edema lesions in the sacroiliac joint occurred infrequently in the asymptomatic population. Moreover, patients presenting with asymptomatic sacroiliitis seemed to have a shorter disease duration than did those with symptomatic sacroiliitis.

**Table 3:** Clinical distribution of articular involvement at enthesitis-related arthritis onset

Articular involved, n (%)	axial ERA (*n* = 57)	peripheral ERA (*n* = 48)	*P*
Shoulder	2 (3.51)	1 (2.08)	1.000
Elbow	3 (5.26)	1 (2.08)	0.737
Wrist	4 (7.02)	2 (4.17)	0.838
Knee	36 (63.16)	27 (56.25)	0.472
Ankle	17 (29.82)	21 (43.75)	0.139
Hip	30 (52.63)	13 (27.08)	0.008*
Cervical	6 (10.53)	0	0.030*
Lumbar	7 (12.28)	0	0.015*

*P＜0.05 is statistically significant

### Drug treatment

Medication history was recorded for both axial and peripheral ERA at onset; at the 6th, 12th, and 18th month; and at the end of follow-up (Table 4). NSAIDs were routinely started as first-line treatment for both peripheral and axial groups. Three patients with the peripheral phenotype were prescribed NSAIDs only at diagnosis, but conventional DMARDs were added within 6 months. The most commonly used conventional DMARDs were MTX (57.89%) for axial ERA and sulfasalazine (66.67%) for peripheral ERA. MTX was also the most widely used co-therapy among patients with ERA using biological DMARDs. The use of biologics varied significantly between the groups from disease onset to the 24th month of follow-up (*P* < 0.05 for all comparisons). The use of DMARDs was less frequent in patients with peripheral than in those with axial ERA. A total of 38 (66.67%) and 10 (20.83%) patients with axial and peripheral ERA, respectively, were treated with biological DMARDs at diagnosis. The most commonly used anti-TNF agent for axial ERA was adalimumab, and etanercept was most commonly used for peripheral ERA. Adalimumab improves axial signs and symptoms in addition to peripheral arthritis and enthesitis.

**Table 4:** Drug treatment in children with enthesitis-related arthritis in the cohort study

Drug treatment, n (%)	axial ERA (*n* = 57)	peripheral ERA (*n* = 48)	*P*
Disease onset			
NSAID	57 (100)	48 (100)	-
DMARD	57 (100)	45 (93.75)	0.184
Biologics	38 (66.67)	10 (20.83)	0.000*
Biologics combined with DMARDs	38 (66.67)	10 (20.83)	0.000*
6-month follow-up			
NSAID	53 (92.30)	45 (93.75)	1.000
DMARD	57 (100)	48 (100)	-
Biologics	39 (68.42)	16 (33.33)	0.000*
Biologics combined with DMARDs	39 (68.42)	16 (33.33)	0.000*
12-month follow-up			
NSAID	53 (92.30)	37 (77.08)	0.020*
DMARD	56 (98.25)	45 (93.75)	0.492
Biologics	39 (68.42)	13 (27.08)	0.000*
Biologics combined with DMARDs	39 (68.42)	13 (27.08)	0.000*
18-month follow-up			
NSAID	43 (75.44)	30 (62.5)	0.151
DMARD	57 (100)	44 (91.67)	0.087
Biologics	41 (71.93)	12 (25)	0.000*
Biologics combined with DMARDs	41 (71.93)	12 (25)	0.000*
End of follow-up			
NSAID	31 (54.39)	22 (45.83)	0.383
DMARD	47 (82.46)	35 (72.92)	0.239
Biologics	31 (54.39)	5 (10.42)	0.000*
Biologics combined with DMARDs	31 (54.39)	4 (8.33)	0.000*

*P＜0.05 is statistically significant

The application of biologics increased gradually in the axial ERA group, peaking at 18 months and decreasing thereafter, whereas that in the peripheral ERA group peaked at 6 months and began to decline thereafter. One patient was successfully treated with secukinumab injection after the failure of TNF-α blockade therapy.

## Discussion

To the best of our knowledge, this was the first systematic analysis of a single-center cohort of axial and peripheral ERA studies in a Chinese population. The study described and compared the clinical characteristics and treatments of patients with axial and those with peripheral ERA. Patients with axial ERA tended to be older, have a longer delay in diagnosis, and exhibit significantly higher levels of inflammatory markers. There were no clear differences in active peripheral joint distribution, except for increased hip arthritis in the axial ERA group. Additionally, patients with axial ERA required more frequent and longer treatment with biologics. These findings highlight the importance of early recognition and identification of axial involvement in ERA.

All of our patients with enthesitis-related, systemic, oligo-, poly-, psoriatic, and undifferentiated JIA were reclassified according to the corresponding ILAR category [1]. Similarly to the previously reported distribution of JIA category in China, ERA was the most common type of JIA in our study cohort, with a prevalence of 34.88% [14]. However, studies from Europe have reported that the most prevalent category of JIA is oligoarthritis, whereas ERA accounts for only 10–16% of all JIA cases [15, 16]. The fact that the epidemiology of JIA in a multiethnic cohort has also shown significant differences in the distribution of JIA subtypes among ethnic groups could explain the different range of values that have been reported [17].

ERA is one of the seven JIA subtypes classified by the ILAR with unique characteristics, including male predominance with later onset, association with HLA-B27, enthesitis, and axial skeleton involvement, in addition to peripheral joint involvement [1]. One pitfall of the ERA category is that it cannot distinguish axial from peripheral phenotypes [8]. Referred to the ASAS criteria, patients with ERA were classified into axial and peripheral groups based on arthritis involvement [10]. The diagnosis of enthesitis/spondylitis-related JIA based on the PRINTO criteria still needs to be validated. The ASAS criteria for peripheral SpA were the most sensitive while ILAR and the preliminary PRINTO criteria were the most specific criteria for classifying ERA patients [18]. In our study, we referred to the ASAS criteria when defined axial and peripheral disease based on arthritis involvement. Our research also show that it is important to develop the classification of ERA for children according to the axis and peripheral involvement. The ineffectiveness of DMARDs in axial disease contributes to the overall poor prognosis for patients with axial ERA [19, 20]. The efficacy of conventional DMARDs on axial disease appears to be less than that on peripheral disease. Increased utilization of MRI has allowed early detection of axial disease, which is sometimes asymptomatic [21, 22]. Anti-TNF agents have been reported to be successful in clinically improving the signs and symptoms of the disease [23–25]. Biologic therapy has variable effects on spinal radiographic progression in patients with axial disease [25]. To achieve a therapeutic response and prevent progressive damage, the axial phenotype of ERA may require more aggressive treatments than the peripheral phenotype.

The most commonly used anti-TNF agents for ERA are etanercept and adalimumab [26, 27]. In our study, more anti-TNF drugs were used in the axial ERA group than in peripheral ERA group from disease onset to the 24th month of follow-up. TNF inhibitors have been an important treatment choice for patients with active axial ERA [25]. A review recently published on this topic determined that no significant difference in spinal radiographic progression was apparent between patients receiving and not receiving TNF inhibitors over the first 2 years; however, after 2 years, a potential protective effect of TNF inhibitors treatment was observed [28]. Although initiated at an early stage in axial disease, the application of biologics still increased gradually over an 18-month period and remained clinically high within the 2-year follow-up. The proportion of biologics used in the peripheral ERA group peaked at 6 months and began to decline thereafter. Furthermore, recently, the American College of Rheumatology has recommended not to use MTX as a monotherapy for children with sacroiliitis [29]. Therefore, treatment for patients with axial ERA should be better tailored, including careful follow-up for disease progression.

In the past, patients with ERA were considered to develop axial involvement only after long periods of inflammation [8]. However, in our study, axial involvement occurred much earlier. According to a recent study, the Assessment in Spondyloarthritis International Society classification criteria for peripheral spondyloarthritis were the most sensitive criteria for classifying patients with ERA and for axial spondyloarthritis criteria, which may aid early detection of axial involvement [18]. During the early stages of the disease, more than half of our patients had axial joint involvement. The reason for this high proportion of patients with axial ERA is that more than one-third of patients with asymptomatic axial ERA were identified and confirmed by available imaging methods [30]. Our study confirmed a high rate of positive MRI findings in such patients at disease onset. Inflammatory and/or erosive lesions of the thoracolumbar spine along with sacroiliitis may exist in the early stage, regardless of the presence of symptoms. Clinical assessment of axial involvement, unlike that of peripheral involvement, can be challenging in the absence of symptoms. The lack of specificity of clinical assessment of axial symptoms is probably explained by the difficult differential diagnosis with other painful conditions, such as fibromyalgia, which may coexist in adults with SpA. In children and adolescents, the presence of thick cartilaginous growth plates may be falsely interpreted as sacroiliitis. Contrast injection may be particularly useful in such cases to show evidence, or not, of inflammation, unlike in adults in which it provides no added value over STIR sequences in MRI of the sacroiliac joints in the early detection of SpA [21, 22, 30]. Application of MRI for suspected axial involvement may be worth considering for ERA, as this may play an important role in preventing the under-determination of axial involvement in patients.

In the present study, there was an average of 10 months of diagnostic delay for patients with axial ERA, which was longer than that for patients with peripheral ERA. Diagnostic delay always contributes to poor radiographic and functional outcome. Moreover, in our study, the axial ERA group was associated with higher ESR levels and more hip joint involvement than was the peripheral ERA group. Elevated ESR is a marker of disease severity and can be used to predict the development of sacroiliac arthritis [7]. Several epidemiological studies of ankylosing spondylitis have reported extra-articular manifestations as the consequences of persistently high levels of inflammation [31].

Hip involvement is a poor prognostic factor for ERA, which increases the risk of sacroiliac arthritis [32, 33]. These various factors could explain why nearly half of our patients with axial ERA were prescribed biological therapy at disease onset. A longer diagnosis delay was correlated with a poor radiographic finding and a higher chance of biological therapy, underscoring the “window of opportunity” concept for JIA treatment. Furthermore, several studies have reported a lack of efficacy of DMARDs in the treatment of axial disease [19, 20]. The axial subtype of ERA is associated with a high mutilation rate and may require an aggressive management approach to achieve a therapeutic response. The lack of recognition of early axial inflammation may lead to delays in provision of appropriate treatment.

Uveitis is a common extra-articular manifestation in patients with ERA. Although the early-onset inflammation is limited to the anterior portion of the eye, the progression of chronic active inflammation may eventually cause significant damage to the posterior pole [34]. Acute anterior uveitis has been reported to be associated with HLA-B27 and ANA [35]. We found a significant increase in the risk for development of uveitis in our patients with ERA who were ANA-positive and female. Interestingly, despite not finding differences in HLA-B27 positivity, ANA positivity, or uveitis incidence between the groups, all five patients with uveitis in this study were both males and HLA-B27-positive. Meanwhile, none of the 18 ANA-positive patients with ERA developed uveitis. This might reflect that ANA has little correlation with uveitis in ERA. The disease characteristics of ERA-associated uveitis are assumed to be different from those of oligoarthritis-associated uveitis [35]. The predominance of male sex and HLA-B27 positivity among patients with uveitis are distinguishing features of ERA, in contrast to those of patients with oligo-related uveitis, which is seen more frequently in female patients, in which ANA positivity predominates and HLA-B27 is not an independent risk factor.

## Conclusions

To date, few studies have focused on the different clinical characteristics and prognosis of axial and peripheral ERA in a systematic manner. Herein, we found that a high proportion of children have axial involvement at the beginning of their diagnosis of ERA and that inflammatory and/or erosive lesions of the sacroiliac joints may exist in asymptomatic patients. Considering that patients with axial ERA tend to have an increased occurrence of delayed diagnosis, higher disease activity, increased likelihood of being exposed to frequent and lengthy treatment with biologics than patients with peripheral ERA, pediatric rheumatologists should consider the treatment of patients with axial disease to be different from that of patients with peripheral ERA. The recognition and assessment of axial arthritis and enthesitis in all possible entheseal sites may have an important implication in the diagnostic and therapeutic approach to ERA. While these findings highlight the importance of classifying the two distinctive disease subtypes of ERA, larger prospective multicenter studies aimed at evaluating the long-term results of the two subgroups are warranted.

## Data Availability

The datasets used and/or analyzed in the current study are available and can be obtained from the corresponding author on reasonable request.

## Abbreviations

ANA: Antinuclear antibody
AS: Ankylosing spondylitis
CRP: C-reactive protein
DMARD: Disease-modifying anti-rheumatic drug
ERA: Enthesitis-related arthritis
ESR: Erythrocyte sedimentation rate
ILAR: International League Against Rheumatism
JIA: Juvenile idiopathic arthritis
MTX: Methotrexate
NSAID: Nonsteroidal anti-inflammatory drug
PRINTO: Pediatric Rheumatology International Trials Organization
RF: Rheumatoid factor
TNF: Tumor necrosis factor
